# Multidisciplinary intervention in obese adolescents: predictors of dropout

**DOI:** 10.1590/S1679-45082015AO3339

**Published:** 2015

**Authors:** Yara Lucy Fidelix, José Cazuza de Farias, Mara Cristina Lofrano-Prado, Ricardo Luís Fernandes Guerra, Michelle Cardel, Wagner Luiz do Prado

**Affiliations:** 1 Universidade de Pernambuco, Recife, PE, Brazil.; 2 Universidade Federal da Paraíba, João Pessoa, PB, Brazil.; 3 Universidade Federal de Pernambuco, Recife, PE, Brazil.; 4 Universidade Federal de São Paulo, Santos, SP Brazil.; 5 University of Colorado, Denver, CO, Estados Unidos.; 6 Universidade Federal de São Paulo, Santos, SP, Brazil;; Programa Associado de Pós-Graduação em Educação Física, Universidade de Pernambuco, Recife, PE, Brazil;; Universidade Federal da Paraíba, João Pessoa, PB, Brazil.

**Keywords:** Patient dropouts, Adiposity, Lipids, Quality of life, Eating disorders, Adolescent

## Abstract

**Objective:**

To identify biological and psychosocial factors associated with dropout in a multidisciplinary behavioral intervention in obese adolescents.

**Methods:**

A total of 183 adolescents (15.4±1.6 years), pubertal (Tanner stage 3 or 4) and obese (34.7±4.0kg/m^2^), were enrolled in a 12-week behavioral intervention, which included clinical consultations (monthly), nutritional and psychological counseling (once a week), and supervised aerobic training (three times/week). The studied variables were weight, height, body mass index, body composition (skinfold), cardiorespiratory fitness (direct gas analysis), blood lipids and self-reported symptoms of eating disorders (bulimia, anorexia and binge eating), anxiety, depression, body image dissatisfaction and quality of life. Statistical analysis included binary logistic regression and independent *t*-tests.

**Results:**

Of the adolescents, 73.7% adhered to the program. The greatest chance for dropout was observed among adolescents older than 15 years (odds ratio of 0.40; 95%CI: 0.15-0.98), with more anorexia symptoms (odds ratio of 0.35; 95%CI: 0.14-0.86) and hypercholesterolemia (odds ratio of 0.40; 95%CI: 0.16-0.91) at baseline.

**Conclusion:**

Older adolescents, with more symptoms of eating disorders and total cholesterol have less chance to adhere to multidisciplinary treatments.

## INTRODUCTION

Evidence suggests that long-term multidisciplinary behavioral intervention is the most effective approach to achieve weight loss and health-related goals,^1^ and that such interventions are more likely to be effective in children and adolescents than adults.^2^

However, the literature indicates a strong relationship between obesity in adolescents and dropout of supervised exercise programs, as well as low rates of success in weight loss programs for overweight/obese adolescents.^3^ Given that adherence has a great influence on weight loss outcomes,^4^ these unexpected results may be at least partly attributed to the high dropout observed in obese children and adolescents.^5^ On average, 50% of adults quit before completing therapy,^4^ whereas in adolescents the dropout rate is 33 to 45%.^6,7^

Several biological and psychosocial barriers make it difficult to adhere to behavioral interventions.^8^ A recent review showed that studies are focusing on the psychological, social and demographic aspects of adherence, but the study of biological factors influencing adherence are limited in the current literature.^9^

Given the high obesity prevalence in adolescents worldwide,^10^ and the well-known deleterious effects of excessive adiposity on health, increasing the risk for premature death,^11^ it is imperative that we study and identify factors related to adherence.^12^ Identification of patients at risk of dropout will contribute to both the efficacy and the cost-effectiveness of weight loss interventions.^13^

## OBJECTIVE

To identify biological and psychosocial factors associated with dropout in a multidisciplinary behavioral intervention in obese adolescents.

## METHODS

### Participants

Obese adolescents were recruited from the greater metropolitan area of the city of Recife, in Brazil, from 2010 to 2013, through local television, newspaper and radio advertisements. The inclusion criteria were: age 12 to 18 years, pubertal stage (3 and 4),^14^ and a body mass index (BMI) >95th percentile for sex and age. Participants were excluded if they had a medical condition that restricted them from engaging in a regular exercise program or if they weighed more than 120kg (due to equipment limitation). Additional exclusion criteria included pregnancy, presence of hypertension or other metabolic complications (hyperinsulinemia and hypercholesterolaemia) and previous use of weight loss drugs. The study was approved by the Ethics Committee of the *Universidade de Pernambuco* (154/09), CAAE: 15798113.9.0000.5207 and the parents/legal guardians signed the Informed Consent Form.

### Study design

A total of 983 adolescents volunteered for the study. During the first visit to the laboratory, pubertal stage, height and weight were measured. In the second visit, the participants performed a rest electrocardiogram, and underwent a medical screening. One hundred eighty-three (183) adolescents met all inclusion criteria and were included in the study. Participants were considered adherent if they participated in at least 75% of all intervention sessions.^15^ All participants received the same multidisciplinary behavioral intervention, as described below.

### Multidisciplinary behavioral intervention

The main goals of the multidisciplinary behavioral intervention were reduction in body weight and promotion of healthy practices and change in lifestyle behaviors. The treatment consisted of clinical, nutritional, psychological and aerobic physical exercise monitoring for 12 weeks, as described elsewhere.^16^ The participants were attended by the same researchers in all cohorts (2010 and 2012: August to October; 2011: March to May; 2013: April to June).

### Endocrinologist consultation

Medical follow-up was performed once a month by an endocrinologist. This included a physical examination to monitor clinical parameters and to facilitate overall compliance with the study.

### Nutritional intervention

Nutritional intervention consisted of group meetings (approximately ten adolescents), once a week, lasting 1 hour. The activities were developed and supervised by a nutritionist. During the meetings, some topics such as fast food, nutritional labeling, types of fat, diet and light products, and strategies for special occasions (holidays, birthdays were addressed) were addressed. Participants were not prescribed any individualized diet.

### Psychotherapy

Psychotherapy was conducted for 1 hour each week in small groups (roughly nine adolescents) by a clinical psychologist. Along with psychological motivation for compliance, the session themes related to body image, eating disorders (symptoms and consequences), the relationship between food and feelings, family and social problems, mood, anxiety and depression were included.

### Supervised aerobic exercise training

Participants performed individualized aerobic training on a treadmill three times a week under the supervision of a physical education professional. The sessions were isocaloric, with energy expenditure fixed at 350kcal/session. Once the training intensity was individualized, the duration of sessions differed between subjects (30 to 60 minutes, approximately).

### Assessment

#### Anthropometrics and body composition

Body mass (kg) was determined using scales (Filizola) with accuracy of 0.1kg and height was measured using a fixed stadiometer with wooden scale accuracy of 0.1cm. Thereafter, we calculated BMI by dividing body weight by height squared (kg/m^2^). The triceps, subscapular and calf skinfolds were measured in the right hemisphere with a caliper Lange^®^ with a resolution of 1mm. The skinfolds were measured in triplicate by the same evaluator, rotational manner, and after mean value was calculated. All measurements followed the protocol and fat mass percentage (%FM) was estimated by the equation.^17^

#### Biochemical analyzes

Sample collection biological material was performed by puncture of peripheral forearm vein after an overnight fast of 12 hours. Blood samples were collected and total cholesterol, high density lipoprotein (HDL), low density lipoprotein (LDL), very low density lipoprotein (VLDL), triglycerides and glucose were determined by immuno-enzymatic assay, using commercial Enzyme Linked Immuno Sorbent Assay (ELISA) kits (Phoenix Pharmaceuticals, Inc. California, United States), following all manufacturer’s recommendations.

#### Cardiorespiratory test

Cardiorespiratory fitness was determined through direct gas analysis during continuous and incremental test on a treadmill (Cosmed T200, Italy). The equipment was calibrated to the gas mixture and volume before each test. The test protocol consisted of 3-minutes warm-up (4km/hour) and 1-minute test was performed increased by 1km/hour, until voluntary exhaustion, or when the Borg scale and the respiratory quotient presented values above 18 and 1.15, respectively.

#### Psychological assessments and quality of life

Adolescents answered questionnaires translated and validated for the Brazilian population. The adolescents were evaluated regarding the presence of eating disorder symptoms (Attitudes Test − EAT-26; Bulimic Investigatory Test Edinburgh – BITE; and Binge Eating Scale − BES), anxiety (State-Trait Anxiety Inventory − STAI), depression (Beck Depression Inventory − BDI), dissatisfaction with body image (Body Shape Questionnaire − BSQ) and self-rated quality of life (Medical Outcomes Study 36 - Item Short - Form Health Survey − SF-36).

## Statistical analysis

The mean difference between groups (adherence *versus* non-adherence) was tested by independent Student’s *t *test. Binary logistic regression was used to analyze association between adherence (people who did adhere = 0 *versus* people who dropout = 1) and the independent variables were age, height, weight, BMI, body composition (fat mass and fat free mass), biochemical analyzes (total cholesterol, LDL, triglycerides, VLDL, HDL, glucose), peak oxygen uptake consumption (peak VO_2_), anorexic symptoms, trait anxiety, state anxiety, depression, binge, bulimia symptoms, bulimia severity, body dissatisfaction and quality of life, all categorized according to the 50th percentile, except for age (<15 years or >15 years), sex (male or female) and training period (morning or afternoon). In the adjusted analysis, the variables with a p value <0.20 for the significance level in the crude analysis were entered into the model and remained in the final model. The a significance level of p≤0.05.

## RESULTS

Out of 183 adolescents included in the sample, 134 (73.2%) adhered to treatment, with no sex differences (76% of boys and 71.3% of girls). Dropout adolescents were older than their adherent counterparts (p=0.01) (Table 1).

**Table 1:** Descriptive table of the baseline age, anthropometric, blood variables, physical fitness, psychological and quality of life in obese adolescents

Variables	Adherence Mean (SD)	Dropout Mean (SD)	p value
Age (years)	15.2 (1.57)	15.9 (1.72)	0.01
Height (m)	1.65 (0.07)	1.63 (0.08)	0.21
Weight (kg)	95.2 (12.4)	92.5 (12.7)	0.20
BMI (kg/m^2^)	34.8 (4.0)	34.5 (4.0)	0.70
Fat mass (%)	51.3 (11.0)	53.0 (6.9)	0.23
Fat mass (kg)	48.7 (13.4)	49.7 (10.0)	0.62
Fat free mass (kg)	45.9 (10.9)	43.7 (8.4)	0.18
Total cholesterol (mg/dL)	167.3 (32.2)	172.8 (38.1)	0.36
LDL (mg/dL)	106.1 (28.8)	106.2 (32.0)	0.99
Triglycerides (mg/dL)	117.7 (68.2)	112.2 (57.2)	0.63
VLDL (mg/dL)	21.0 (12.8)	20.9 (8.8)	0.97
Glucose (mg/dL)	83.8 (11.9)	83.4 (9.5)	0.86
HDL (mg/dL)	49.0 (9.3)	52.0 (69.9)	0.22
Peak VO_2_ (mL/kg/min)	25.7 (4.4)	26.6 (5.4)	0.39
Anorexic symptoms	19.2 (8.9)	21.9 (10.9)	0.15
Trait anxiety	45.6 (10.0)	45.1 (12.0)	0.78
Depression	17.3 (8.8)	17.8 (9.1)	0.77
State-anxiety	40.7 (9.8)	41.9 (10.5)	0.64
Binge	13.7 (7.6)	14.5 (8.9)	0.59
Bulimia symptoms	13.5 (5.3)	15.4 (6.6)	0.09
Bulimia severity	2.2 (2.5)	2.5 (3.2)	0.57
Body dissatisfaction	119.1 (32.1)	121.9 (37.4)	0.59
Physical functioning	71.1 (21.8)	71.7 (23.0)	0.89
Physical role	72.0 (30.1)	77.0 (29.9)	0.41
Pain	72.5 (22.1)	70.4 (19.9)	0.63
General health perception	60.8 (23.7)	57.4 (22.5)	0.47
Vitality	60.1 (21.6)	62.3 (23.9)	0.61
Social functioning	78.7 (21.6)	76.3 (26.5)	0.59
Emotional role	68.9 (35.2)	74.4 (35.8)	0.44
Mental health	70.6 (22.7)	72.6 (23.6)	0.66
Mean of dimensions	69.5 (17.1)	70.7 (17.9)	0.72

Independent Student’s *t* test. SD: standard deviation; BMI: body mass index; LDL: low density lipoprotein; VLDL: very low density lipoprotein; HDL: high density lipoprotein; peak VO_2_: peak oxygen consumption.

In the crude analysis we observed that age (odds ratio of 0.48; confidence interval 95%CI: 0.24-0.97) was associated with dropout, showing that adolescents aged >15 years had 0.48 less chance to adhere to the program, compared to younger ones (Table 2).

**Table 2:** Crude analysis of factors associated with dropout in a multidisciplinary program for the treatment of obesity in adolescents

Variables*	Odds ratio	p value	95%CI
Sex	0.78	0.48	0.40-1.54
Shift	1.31	0.45	0.64-2.68
Age (years)	0.48	<0.01	0.24-0.97
Anorexic symptoms	0.50	0.09	0.22-1.13
Peak VO_2_ (mL/kg/min)	1.25	0.54	0.60-2.56
Bulimia symptoms	0.82	0.63	0.37-1.82
Binge	0.97	0.94	0.44-2.14
Body dissatisfaction	1.00	1.00	0.45-2.21
Bulimia severity	0.60	0.21	0.27-1.33
Trait-anxiety	1.21	0.63	0.55-2.69
State-anxiety	0.79	0.66	0.28-2.55
Depression	0.83	0.63	0.37-1.82
Total cholesterol (mg/dL)	0.58	0.12	0.28-1.17
HDL (mg/dL)	0.88	0.72	0.44-1.77
LDL (mg/dL)	0.94	0.88	0.44-2.03
VLD-L (mg/dL)	0.58	0.25	0.23-1.47
Glucose (mg/dL)	0.95	0.89	0.48-1.90
Triglycerides (mg/dL)	0.81	0.55	0.40-1.64
Physical functioning	0.59	0.19	0.26-1.30
Physical role	1.23	0.61	0.55-2.74
Pain	1.54	0.28	0.69-3.46
General health perception	1.46	0.35	0.66-3.23
Vitality	0.73	0.44	0.33-1.63
Social functioning	0.84	0.66	0.38-1.86
Emotional role	0.53	0.12	0.23-1.19
Mental health	1.03	0.94	0.47-2.27

Binary logistic regression. *The categories of reference were always below the 50th percentile, sex = male, shift = morning, age: <15 years. 95%CI: 95% confidence interval; peak VO_2_ = peak oxygen consumption; HDL: high density lipoprotein; LDL: low density lipoprotein; VLD-L: very low density lipoprotein.

The adjusted analysis (Table 3) showed that adolescents aged over 15 years and those above the 50th percentile for scores of anorexia and total cholesterol were less chance to adhere to the program. The other variables were not associated with dropout.

**Table 3:** Adjusted analysis of factors associated with dropout in a multidisciplinary program for the treatment of obesity in adolescents

Variables*	Odds ratio	p value	95%CI
Age (years)	0.40	0.05	0.15-0.98
Anorexic symptoms	0.35	0.02	0.14-0.86
Total cholesterol (mg/dl)	0.40	0.05	0.16-0.91
Physical functioning	0.67	0.41	0.26-1.73
Emotional role	0.47	0.13	0.18-1.26

Binary logistic regression. * The categories of reference were always below the 50th percentile and age = <15 years. 95%CI: 95% confidence interval.

## DISCUSSION

The main findings of this study were: adherence of 73.2% and lower chance of treatment adherence in adolescents aged over 15 years, with higher scores for symptoms of anorexia and total cholesterol.

Studies relating adherence to behavior changes showed that 60 to 80% of individuals reached the minimum percentage of prescribed sessions, and about half of the participants dropped out in the first 6 months of treatment, even before the health benefits of treatment are observed.^9^ The estimated proportion of individuals that drop out from programs including exercise and changes in lifestyle is approximately 50%.^4,18^ However, studies have not used multidisciplinary approaches and supervised exercises,^7^ and this may explain the greater adherence observed in the present study. Furthermore, it is believed that the short duration of the intervention (12 weeks), may have contributed to this increased adherence to treatment. The different populations studied (elderly, individuals with chronic diseases, adults) and the methodology used in the studies limit comparisons of results.

The literature suggests that patients with chronic diseases, which includes obesity, have lower adherence to treatment, since the therapies, often complex, require some dedication and the patient should be followed up continuously.^19^ A study examined for 2 consecutive years the follow-up of obese children and adolescents in specialized clinics, which provide medical, nutritional, psychological and physical evaluations, and found that patient return visits gradually decreased, and 43% of them dropped out.^20^

The therapies that aim to change behavior provide patients with cognitive and behavioral skills to modify the lifestyle, helping people realize that adopting an active lifestyle and healthy life is a positive opportunity for a new life, beyond weight control in the lon0g term.^12^ However, behavioral changes related to the nutritional aspect, which is also part of the multidisciplinary program of this study, seems difficult to achieve, since some studies showed low rates of adherence to treatments with nutritional interventions.^21,22^ Independent of the type of nutrition counseling proposed (individual or group), the change in eating habits is still unsatisfactory, and studies that assess adherence to this type of treatment should be performed to constantly improve care of chronic disease patients.^21^

Our data demonstrates that higher scores of anorexia symptoms are inversely associated with compliance in a program to treat obesity, thus showing the importance of including psychological assessments in obese individuals prior to treatment. The literature suggests that individuals with symptoms of anorexia and receiving behavioral therapy, which was also used in this study, are more likely to drop out treatment than those receiving non-specific approaches.^23^ Moreover, the literature indicates that individuals with severe anorexic symptoms present problems to recover adipose tissue.^24,25^We believe that this may hinder the perception of changes in the body, leading to demotivation and, consequently, treatment dropout.

The literature shows some variables that are associated with dropout in programs aiming at weight loss. These variables include not very realistic expectations, which are associated to greater disappointment and, consequently, treatment dropout,^12^ females; individuals with a higher BMI; those who reported previous attempts at weight loss; and younger age groups.^26^ In relation to age, the literature indicates a negative relation with physical exercise and healthy habits.^27^ In the present study, we observed that older adolescents are more likely to drop out treatment as compared to younger ones. It is common for parents of younger adolescents to accompany them to the intervention, decreasing the chance of dropping out. This hypothesis is based on the Theory of Social Control, which states that when adolescents are close to their parents, they tend to behave to please the adults.^28^

This study found no association between adherence and anthropometric indicators, unlike the study TIGER (Training Interventions and Genetics of Exercise Response), which identified that individuals with lower values of body mass, waist circumference, hip and BMI, adhered more to the aerobic exercise program.^29^ The difference between the results may be due to the type of study, beyond the age investigated, since the TIGER is an epidemiological study of adults (18 to 35 years). The present study addressed adolescents, and homogeneity of the participants’ characteristics is an important factor to be considered. This study found an association between adherence and high levels of cholesterol; nonetheless, further studies are required to prove this hypothesis, since the literature has not demonstrated yet a plausible relation. Studies that investigate variables associated with biological motivation for adherence are still very scarce,^9^ thus preventing comparison of the results. We also emphasize the importance of further studies in this area, because most adolescents that should be treated to reduce lipid levels were precisely those who did not complete the intervention. The present study has some limitations, including the use of self-reported questionnaires, which may have compromised veracity due to recall bias in some cases, and, as the subjects were volunteers, only those who were willing to participate engaged in the intervention. The absence of family history data should be considered in the interpretation of the present data. Moreover, because it is a cross-sectional study it was not possible to establish a cause and effect relation between variables.

Finally, we verified that no single variable is able to explain and/or predict dropout in treatment programs for obesity, confirming the continuing need to investigate how the independent effects and interrelations between biological, psychological and environmental factors may influence adherence.^30^ In order to increase compliance in multidisciplinary programs for obesity treatment, a better understanding of predisposing healthy or risk behaviors, resulting from interaction of biological, psychological, environmental and genetic factors is needed.^9^

## CONCLUSION

The results of this study provided evidence that older adolescents, with more symptoms of eating disorders and total cholesterol have less chance to adhere to multidisciplinary treatments weight loss.
