# Supplementary material for: Multidisciplinary intervention in obese adolescents: predictors of dropout
Source: Einstein (Sao Paulo). 2015 Jul-Sep;13(3):388–94. doi: 10.1590/S1679-45082015AO3339 (PMC4943784; doi:10.1590/S1679-45082015AO3339)
Supplement: Translation

# Translation: Intervenção multidisciplinar em adolescentes obesos: preditores de desistência

**Authors:** Yara Lucy Fidelix, José Cazuza de Farias, Mara Cristina Lofrano-Prado, Ricardo Luís Fernandes Guerra, Michelle Cardel, Wagner Luiz do Prado

**Affiliations:** 1 Universidade de Pernambuco, Recife, PE, Brasil.; 2 Universidade Federal da Paraíba, João Pessoa, PB, Brasil.; 3 Universidade Federal de Pernambuco, Recife, PE, Brasil.; 4 Universidade Federal de São Paulo, Santos, SP, Brasil.; 5 University of Colorado, Denver, CO, Estados Unidos.; 6 Universidade Federal de São Paulo, Santos, SP, Brasil;; Programa Associado de Pós-Graduação em Educação Física, Universidade de Pernambuco, Recife, PE, Brasil;; Universidade Federal da Paraíba, João Pessoa, PB, Brasil.

**Keywords:** Pacientes desistentes do tratamento, Adiposidade, Lipídeos, Qualidade de vida, Transtornos da alimentação, Adolescente

## Abstract

**Objetivo:**

Identificar os fatores biológicos e psicossociais associados à desistência de uma intervenção multidisciplinar comportamental em adolescentes obesos.

**Métodos:**

Foram selecionados para participar das 12 semanas de intervenção 183 adolescentes (15,4±1,6 anos), púberes (Tanner 3 ou 4) e obesos (34,7±4,0 kg/m^2^). A intervenção incluiu consultas clínicas (mensal), aconselhamento nutricional e psicológico (uma vez/semana), e treinamento aeróbio supervisionado (três vezes/semana). As variáveis estudadas foram peso, altura, índice de massa corporal, composição corporal (dobras cutâneas), aptidão cardiorrespiratória (análise direta de gases), perfil lipídico e sintomas autorrelatados de transtornos alimentares (bulimia, anorexia e compulsão alimentar), ansiedade, depressão, insatisfação com a imagem corporal e qualidade de vida. A análise estatística incluiu regressão logística binária e teste *t *de Student independente.

**Resultados:**

Aderiram ao programa 73,7% dos adolescentes. A maior chance de desistência foi observada entre aqueles com mais de 15 anos (*odds ratio* de 0,40; IC95%: 0,15-0,98), com mais sintomas de anorexia (*odds ratio* de 0,35; IC95%: 0,14-0,86) e com hipercolesterolemia (*odds ratio* de 0,40; IC95%: 0,16-0,91) no início do estudo.

**Conclusão:**

Adolescentes mais velhos, com mais sintomas de transtornos alimentares e de níveis de colesterol total, têm menor chance de aderir a tratamentos multidisciplinares.

## INTRODUÇÃO

Existem evidências de que a intervenção comportamental multidisciplinar de longo prazo é a abordagem mais eficiente para alcançar a perda de peso e outros objetivos relativos à saúde,^1^ e que essas intervenções têm maior probabilidade de sucesso em crianças e adolescentes do que em adultos.^2^

No entanto, a literatura sugere uma forte relação entre a obesidade em adolescentes e a desistência de programas de exercícios supervisionados, bem como baixas taxas de sucesso em programas de perda de peso para adolescentes com sobrepeso/obesos.^3^ Considerando-se que a adesão exerce grande influência nos resultados de perda de peso,^4^ esses resultados inesperados podem ser, no mínimo, parcialmente atribuídos à alta desistência observada em crianças e adolescentes obesos.^5^ Em média, 50% dos adultos desistem antes da conclusão do tratamento,^4^ enquanto em adolescentes a taxa de desistência é de 33 a 45%.^6,7^

Diversas barreiras biológicas e psicossociais dificultam a adesão às intervenções comportamentais.^8^ Uma revisão recente demonstrou que os estudos se concentram nos aspectos psicológicos, sociais e demográficos da adesão e que são poucos os estudos na literatura atual sobre os fatores biológicos que influenciam na adesão.^9^

Considerando-se a alta prevalência mundial de obesidade em adolescentes,^10^ e os efeitos nocivos bem conhecidos da adiposidade excessiva na saúde, que levam ao aumento do número de morte prematura,^11^ é muito importante estudar e identificar os fatores relacionados à adesão.^12^ A identificação dos pacientes com risco de desistência contribuirá com a eficácia e a custo-efetividade das intervenções para perda de peso.^13^

## OBJETIVO

Identificar os fatores biológicos e psicossociais associados à desistência em uma intervenção comportamental multidisciplinar para adolescentes obesos.

## MÉTODOS

### Participantes

Adolescentes obesos foram recrutados na área metropolitana da grande Recife, no Brasil, de 2010 a 2013, por meio de anúncios em canais de televisão, jornais e rádios locais. Os critérios de inclusão foram: 12 a 18 anos, estágio púbere (3 e 4),^14^ e índice de massa corporal (IMC) > percentil 95 de sexo e idade. Os participantes foram excluídos caso tivessem qualquer doença que os impedisse de participar de um programa regular de exercícios, ou pesassem mais de 120kg (devido a limitações dos equipamentos). Outros critérios de exclusão incluíram gravidez, presença de hipertensão ou complicações metabólicas (hiperinsulinemia e hipercolesterolemia), e uso prévio de medicamentos para perda de peso. O estudo foi aprovado pelo Comitê de Ética da Universidade de Pernambuco (154/09), CAAE: 15798113.9.0000.5207 e foi obtido Consentimento Livre e Esclarecido por escrito dos participantes e dos pais/responsáveis legais.

### Desenho do estudo

No total, 983 adolescentes se voluntariaram para este estudo. Na primeira visita ao laboratório, foram medidos o estágio púbere, a altura e o peso. Na segunda visita, os participantes foram submetidos a um eletrocardiograma de repouso e uma avaliação médica. Cento e oitenta e três (183) adolescentes cumpriram todos os critérios de inclusão e foram, portanto, incluídos no estudo. Os participantes foram considerados aderentes quando tivessem participado de pelo menos 75% de todas as sessões da intervenção.^15^ Todos os participantes receberam a mesma intervenção comportamental multidisciplinar, conforme descrito a seguir.

### Intervenção comportamental multidisciplinar

Os principais objetivos da intervenção comportamental multidisciplinar foram a redução do peso corporal e a promoção de práticas saudáveis e mudanças nos comportamentos relativos ao estilo de vida. O tratamento incluiu monitoramento clínico, nutricional, psicológico e de exercícios físicos aeróbicos por 12 semanas, conforme já descrito em outra ocasião.^16^ Os participantes foram atendidos pelos mesmos pesquisadores em todas as coortes (2010 e 2012: agosto a outubro; 2011: março a maio; 2013: abril a junho).

### Consulta com endocrinologista

O acompanhamento médico foi realizado uma vez por mês, por um endocrinologista. Essa consulta incluía um exame físico para monitorar parâmetros clínicos e para facilitar a adesão às regras do estudo em geral.

### Intervenção nutricional

A intervenção nutricional consistiu em reuniões em grupo (aproximadamente dez adolescentes), uma vez por semana, durante 1 hora. As atividades foram desenvolvidas e supervisionadas por um nutricionista. Durante as reuniões, temas como *fast food*, rótulos de alimentos, tipos de gordura, produtos *diet* e *light*, e estratégias para ocasiões especiais (férias e aniversários) foram abordados. Não foram prescritas dietas personalizadas para os participantes.

### Psicoterapia

Sessões de psicoterapia em pequenos grupos (cerca de nove adolescentes) foram realizadas por um psicólogo clínico durante 1 hora por semana. Além da motivação psicológica para adesão à intervenção, as sessões incluíram temas relativos à imagem corporal, distúrbios alimentares (sintomas e consequências), relação entre a comida e os sentimentos, problemas familiares e sociais, humor, ansiedade e depressão.

### Treinamento aeróbico supervisionado

Os participantes realizaram treinamento aeróbico individual em uma esteira, três vezes por semana, sob supervisão de um profissional de educação física. As sessões foram isocalóricas, com gasto de energia fixado em 350kcal/sessão. Depois que a intensidade do treinamento foi individualizada, a duração das sessões variou entre os participantes (30 a 60 minutos, aproximadamente).

### Avaliação

#### Antropometria e composição corporal

A massa corporal (kg) foi determinada em uma balança (Filizola) com precisão de 0,1kg, e a altura foi medida utilizando-se um estadiômetro fixo com escala de madeira e precisão de 0,1cm. Depois, calculou-se o IMC, dividindo-se o peso corporal pela altura ao quadrado (kg/m^2^). As dobras cutâneas triciptal, subescapular e da panturrilha foram medidas no hemisfério direito utilizando-se um adipômetro Lange^®^ com resolução de 1mm. As dobras cutâneas foram medidas em triplicata, com o mesmo medidor e em rodízio, e o valor médio foi calculado. Todas as medições seguiram o protocolo e a porcentagem de gordura corporal (%FM) foi estimada pela equação.^17^

#### Análises bioquímicas

A coleta de amostras de material biológico foi realizada com punção de veia periférica do antebraço, após jejum noturno de 12 horas. Amostras de sangue foram coletadas e foram determinados os níveis de colesterol total, lipoproteína de alta densidade (HDL), lipoproteína de baixa densidade (LDL), lipoproteína de densidade muito baixa (VLDL), triglicerídeos e glicose, utilizando-se um ensaio imunoenzimático e *kits* comerciais de *EnzymeLinked Immuno Sorbent Assay* (ELISA; Phoenix Pharmaceuticals, Inc. Califórnia, Estados Unidos), de acordo com as recomendações do fabricante.

#### Teste de esforço cardiorrespiratório

O condicionamento cardiorrespiratório foi determinado por uma análise direta de gases, durante um teste contínuo e incremental em uma esteira (Cosmed T200, Itália). O equipamento foi calibrado para a mistura e o volume de gases antes de cada teste. O protocolo do teste consistia em um aquecimento de 3 minutos (4km/h), e um teste a cada minuto, com incremento de 1km/h, até a exaustão voluntária, ou até que os valores da escala de Borg e de quociente respiratório ultrapassassem 18 e 1,15, respectivamente.

#### Avaliações psicológicas e de qualidade de vida

Os adolescentes responderam a questionários traduzidos e validados para a população brasileira. Eles foram avaliados quanto à presença de sintomas de distúrbios alimentares (Teste de Atitudes − EAT-26; Teste de Investigação Bulímica de Edimburgo (BITE – *Bulimic Investigatory Test Edinburgh*); e Escala de Compulsão Alimentar Periódica − ECAP), ansiedade (Inventário de Ansiedade Traço-Estado − IDATE), depressão (Inventário de Depressão de Beck (BDI – *Beck Depression Inventory*), insatisfação com a imagem corporal (Questionário de Imagem Corporal (BSQ – *Body Shape Questionnaire*) e qualidade de vida autoavaliada (*Medical Outcomes Study 36 - Item Short - Form Health Survey* − SF-36).

## Análise estatística

A diferença média entre os grupos (aderentes *versus* não aderentes) foi avaliada com o teste *t *de Student independente. A regressão logística binária foi usada para analisar a associação entre a adesão (pessoas que aderiram = 0 *versus* pessoas que desistiram = 1) e as variáveis independentes foram idade, altura, peso, IMC, composição corporal (massa de gordura e massa magra), análises bioquímicas (colesterol total, LDL, triglicerídeos, VLDL, HDL, glicose), consumo máximo de oxigênio (VO_2_ máximo), sintomas de anorexia, ansiedade-traço, ansiedade-estado, depressão, compulsão alimentar periódica, sintomas de bulimia, gravidade da bulimia, insatisfação com o corpo e qualidade de vida, todas categorizadas de acordo com o percentil 50, exceto a idade (<15 anos ou >15 anos), o sexo (masculino ou feminino) e o período de treinamento (manhã ou tarde). Na análise ajustada, as variáveis com valor de p<0,20 para o nível de significância na análise bruta foram inseridas no modelo e permaneceram no modelo final. O nível de significância adotado foi p≤0,05.

## RESULTADOS

Do total de 183 adolescentes incluídos na amostra, 134 (73,2%) aderiram ao tratamento, sem diferenças entre os sexos (76% para os meninos e 71,3% para as meninas). Os adolescentes desistentes foram mais velhos que os aderentes (p=0,01) (Tabela 1).

**Tabela 1:** Tabela descritiva da idade basal, das medidas antropométricas, das variáveis sanguíneas, do condicionamento físico, dos fatores psicológicos e da qualidade de vida em adolescentes obesos

Variáveis	Aderente Média (DP)	Desistente Média (DP)	Valor de p
Idade (anos)	15,2 (1,57)	15,9 (1,72)	0,01
Altura (m)	1,65 (0,07)	1,63 (0,08)	0,21
Peso (kg)	95,2 (12,4)	92,5 (12,7)	0,20
IMC (kg/m^2^)	34,8 (4,0)	34,5 (4,0)	0,70
Massa de gordura (%)	51,3 (11,0)	53,0 (6,9)	0,23
Massa de gordura (kg)	48,7 (13,4)	49,7 (10,0)	0,62
Massa magra (kg)	45,9 (10,9)	43,7 (8,4)	0,18
Colesterol total (mg/dL)	167,3 (32,2)	172,8 (38,1)	0,36
LDL (mg/dL)	106,1 (28,8)	106,2 (32,0)	0,99
Triglicerídeos (mg/dL)	117,7 (68,2)	112,2 (57,2)	0,63
VLDL (mg/dL)	21,0 (12,8)	20,9 (8,8)	0,97
Glicose (mg/dL)	83,8 (11,9)	83,4 (9,5)	0,86
HDL (mg/dL)	49,0 (9,3)	52,0 (69,9)	0,22
VO_2_ máximo (mL/kg/min)	25,7 (4,4)	26,6 (5,4)	0,39
Sintomas de anorexia	19,2 (8,9)	21,9 (10,9)	0,15
Ansiedade-traço	45,6 (10,0)	45,1 (12,0)	0,78
Depressão	17,3 (8,8)	17,8 (9,1)	0,77
Ansiedade-estado	40,7 (9,8)	41,9 (10,5)	0,64
Compulsão alimentar periódica (Binge)	13,7 (7,6)	14,5 (8,9)	0,59
Sintomas de bulimia	13,5 (5,3)	15,4 (6,6)	0,09
Gravidade da bulimia	2,2 (2,5)	2,5 (3,2)	0,57
Insatisfação com o corpo	119,1 (32,1)	121,9 (37,4)	0,59
Funcionamento físico	71,1 (21,8)	71,7 (23,0)	0,89
Papel do físico	72,0 (30,1)	77,0 (29,9)	0,41
Dor	72,5 (22,1)	70,4 (19,9)	0,63
Percepção de saúde geral	60,8 (23,7)	57,4 (22,5)	0,47
Vitalidade	60,1 (21,6)	62,3 (23,9)	0,61
Funcionamento social	78,7 (21,6)	76,3 (26,5)	0,59
Papel do emocional	68,9 (35,2)	74,4 (35,8)	0,44
Saúde mental	70,6 (22,7)	72,6 (23,6)	0,66
Média das dimensões	69,5 (17,1)	70,7 (17,9)	0,72

Teste* t* de Student independente. DP: desvio padrão; IMC: índice de massa corporal; LDL: lipoproteína de baixa densidade; VLDL: lipoproteína de densidade muito baixa; HDL: lipoproteína de alta densidade; VO_2 _máximo: consumo máximo de oxigênio.

Na análise bruta, observaram-se que a idade (*oddsratio* de 0,48; intervalo de confiança de IC95%: 0,24- 0,97) foi associada à desistência, já que adolescentes >15 anos apresentaram chance 0,48 menor de aderir ao programa, em comparação aos mais novos (Tabela 2).

**Tabela 2:** Análise bruta de fatores associados à desistência em um programa multidisciplinar para tratamento de obesidade em adolescentes

Variáveis*	*Odds ratio*	Valor de p	IC95%
Sexo	0,78	0,48	0,40-1,54
Turno	1,31	0,45	0,64-2,68
Idade (anos)	0,48	<0,01	0,24-0,97
Sintomas de anorexia	0,50	0,09	0,22-1,13
VO_2_ máximo (mL/kg/min)	1,25	0,54	0,60-2,56
Sintomas de bulimia	0,82	0,63	0,37-1,82
Compulsão alimentar periódica (Binge)	0,97	0,94	0,44-2,14
Insatisfação com o corpo	1,00	1,00	0,45-2,21
Gravidade da bulimia	0,60	0,21	0,27-1,33
Ansiedade-traço	1,21	0,63	0,55-2,69
Ansiedade-estado	0,79	0,66	0,28-2,55
Depressão	0,83	0,63	0,37-1,82
Colesterol total (mg/dL)	0,58	0,12	0,28-1,17
HDL (mg/dL)	0,88	0,72	0,44-1,77
LDL (mg/dL)	0,94	0,88	0,44-2,03
VLD-L (mg/dL)	0,58	0,25	0,23-1,47
Glicose (mg/dL)	0,95	0,89	0,48-1,90
Triglicerídeos (mg/dL)	0,81	0,55	0,40-1,64
Funcionamento físico	0,59	0,19	0,26-1,30
Papel do físico	1,23	0,61	0,55-2,74
Dor	1,54	0,28	0,69-3,46
Percepção de saúde geral	1,46	0,35	0,66-3,23
Vitalidade	0,73	0,44	0,33-1,63
Funcionamento social	0,84	0,66	0,38-1,86
Papel do emocional	0,53	0,12	0,23-1,19
Saúde mental	1,03	0,94	0,47-2,27

Regressão logística binária. *As categorias de referência foram estar abaixo do percentil 50, sexo masculino, turno da manhã e idade <15 anos. IC95%: intervalo de confiança de 95%; VO_2_ máximo: consumo máximo de oxigênio; HDL: lipoproteína de alta densidade; LDL: lipoproteína de baixa densidade; VLD-L: lipoproteína de densidade muito baixa.

A análise ajustada (Tabela 3) demonstrou que os adolescentes com mais de 15 anos, e aqueles acima do percentil 50 para os escores de anorexia e colesterol total apresentaram menor chance de aderir ao programa.As outras variáveis não foram associadas à desistência.

**Tabela 3:** Análise ajustada de fatores associados à desistência em um programa multidisciplinar para tratamento de obesidade em adolescentes

Variáveis*	*Odds ratio*	Valor de p	IC95%
Idade (anos)	0,40	0,05	0,15-0,98
Sintomas de anorexia	0,35	0,02	0,14-0,86
Colesterol total (mg/dL)	0,40	0,05	0,16-0,91
Funcionamento físico	0,67	0,41	0,26-1,73
Papel do emocional	0,47	0,13	0,18-1,26

Regressão logística binária. *As categorias de referência foram estar abaixo do percentil 50 e idade <15 anos. IC95%: Intervalo de confiança de 95%.

## DISCUSSÃO

Os principais achados do estudo foram: adesão de 73,2% e menor chance de adesão ao tratamento em adolescentes acima de 15 anos, com escores mais altos para sintomas de anorexia e colesterol total.

Estudos relacionados à adesão para mudança de comportamento demonstram que 60 a 80% dos indivíduos atingiram a porcentagem mínima de sessões indicadas, e cerca de metade dos pacientes desistem nos primeiros 6 meses de tratamento, mesmo antes de se observarem os benefícios de saúde previstos.^9^ A proporção estimada de indivíduos que desistem de programas que incluam exercícios e mudanças no estilo de vida é de aproximadamente 50%.^4,18^ No entanto, esses estudos não utilizaram abordagens multidisciplinares, nem exercícios supervisionados,^7^ e isso poderia explicar a maior adesão observada no presente estudo. Além disso, acredita-se que a curta duração da intervenção (12 semanas) pode ter contribuído para o aumento da adesão ao tratamento. As diferentes populações estudadas (idosos, indivíduos com doenças crônicas, adultos) e a metodologia utilizada nos estudos limitam as comparações dos resultados.

A literatura sugere que os pacientes com doenças crônicas, inclusive obesidade, apresentam uma adesão mais baixa ao tratamento, já que as terapias, muitas vezes complexas, requerem dedicação e acompanhamento contínuo do paciente.^19^ Um estudo analisou um período de 2 anos consecutivos de acompanhamento de crianças e adolescentes obesos em clínicas especializadas, que oferecem avaliações médicas, nutricionais, psicológicas e físicas, e demonstrou que os retornos dos pacientes diminuíam gradativamente e que 43% desistiram do tratamento.^20^

As terapias para mudança de comportamento equipam os pacientes com competências cognitivas e comportamentais para que possam mudar seu estilo de vida, ajudando-os a enxergar que a adoção de um estilo de vida ativo e saudável é uma boa oportunidade de começar uma vida nova, indo além do controle do peso no longo prazo.^12^ No entanto, as mudanças comportamentais relativas ao aspecto nutricional, que também fazem parte do programa multidisciplinar deste estudo, parecem mais difíceis de serem alcançadas, já que os estudos demonstram baixas taxas de adesão aos tratamentos com intervenções nutricionais.^21,22^ Independentemente do tipo de aconselhamento nutricional proposto (individual ou em grupo), a mudança dos hábitos alimentares permanece insatisfatória, e estudos para avaliar a adesão a esse tipo de tratamento devem ser realizados para melhorar continuamente o atendimento aos pacientes com doenças crônicas.^21^

Nossos dados demonstram que escores mais altos de sintomas de anorexia estiveram inversamente associados à adesão a este programa para tratamento de obesidade, e isso mostrou a importância da realização de avaliações psicológicas com indivíduos obesos antes do tratamento. A literatura indica que pacientes com sintomas de anorexia submetidos à terapia comportamental, que também foi usada neste estudo, têm maior chance de desistir do tratamento que aqueles tratados com abordagens não específicas.^23^ Além disso, a literatura sugere que os pacientes com sintomas graves de anorexia apresentam problemas na restauração do tecido adiposo.^24,25^ Acreditamos que isso possa atrapalhar a percepção de mudanças corporais, levando à desmotivação e, consequentemente, à desistência do tratamento.

A literatura mostra que algumas variáveis estão associadas à desistência em programas destinados principalmente à perda de peso. Essas variáveis incluem expectativas pouco realistas, associadas a um maior nível de desapontamento e, consequentemente, desistência do tratamento,^12^ sexo feminino; indivíduos com IMC mais alto; pacientes que reportam tentativas anteriores de perda de peso; e faixas etárias mais jovens.^26^ Em relação à idade, a literatura aponta para uma relação negativa com exercícios físicos e hábitos saudáveis.^27^ Neste estudo, observamos que adolescentes mais velhos tiveram menor chance de aderir ao tratamento, em comparação aos mais novos. É comum que os pais de adolescentes mais jovens os acompanhem às sessões, o que reduz a chance de abandono. Essa hipótese baseia-se na Teoria do Controle Social, que afirma que quando os adolescentes estão com os pais, eles se sentem inclinados a agir de forma que os agrade.^28^

Este estudo não detectou nenhuma associação entre a adesão e os indicadores antropométricos, diferentemente do estudo TIGER (*Training Interventions and Genetics of Exercise Response*), que identificou que os indivíduos com valores mais baixos de massa corporal, circunferência abdominal, quadril e IMC são mais aderentes aos programas de exercícios aeróbicos.^29^ A diferença entre os resultados pode ser explicada pelo tipo de estudo, além da idade pesquisada, já que o TIGER foi um estudo epidemiológico realizado em adultos (18 a 35 anos), e este foi um estudo clínico em adolescentes, no qual a homogeneidade das características dos participantes é um fator importante a ser considerado. Este estudo detectou uma associação entre a adesão e altos níveis de colesterol, mas são necessários mais estudos para comprovar essa hipótese, já que não há nada na literatura que demonstre a plausibilidade dessa relação.Os estudos que investigaram as variáveis associadas à motivação biológica da adesão são escassos,^9^ e isso impede a comparação dos resultados. Destacamos também a importante necessidade de estudos adicionais nessa área, já que a maioria dos adolescentes que deveriam ser tratados para redução dos níveis de colesterol total são exatamente os que não concluíram o tratamento. Este estudo apresenta algumas limitações, como o uso de questionários autorreportados, que podem ter comprometido a veracidade devido ao viés da reconvocação em alguns casos e, como os indivíduos eram voluntários, apenas aqueles dispostos a participar é que efetivamente se envolveram na intervenção. A ausência de informações de histórico familiar deve ser considerada na interpretação destes dados. Além disso, como se trata de um estudo transversal, não foi possível estabelecer uma relação de causa e efeito entre as variáveis.

Finalmente, verificamos que nenhuma variável isolada conseguiria explicar e/ou prever a desistência dos programas para tratamento de obesidade, o que confirma a necessidade de se continuar investigando como os efeitos independentes e as inter-relações entre os fatores biológicos, psicológicos e ambientais podem influenciar na adesão.^30^ Para aumentar a adesão em programas multidisciplinares para o tratamento da obesidade, é preciso compreender melhor a predisposição a comportamentos saudáveis ou de risco, que resultam da interação de fatores biológicos, psicológicos, ambientais e genéticos.^9^

## CONCLUSÃO

Os resultados deste estudo forneceram evidências de que, adolescentes mais velhos, com maiores sintomas de distúrbios alimentares e de níveis de colesterol total, têm menor chance de aderir a tratamentos multidisciplinares para perda de peso.
